# Barriers and Facilitators to the Adoption of Mobile Health Among Health Care Professionals From the United Kingdom: Discrete Choice Experiment

**DOI:** 10.2196/17704

**Published:** 2020-07-06

**Authors:** Simon Leigh, Liz Ashall-Payne, Tim Andrews

**Affiliations:** 1 The Organisation for the Review of Care and Health Applications Daresbury United Kingdom

**Keywords:** digital health, mHealth, discrete-choice, preferences, mobile phone

## Abstract

**Background:**

Despite the increasing availability of mobile health services, clinical engagement remains minimal.

**Objective:**

This study aims to identify and weight barriers to and drivers of health app use among health care professionals (HCPs) from the United Kingdom.

**Methods:**

A discrete choice experiment was conducted with 222 HCPs using a web-based survey between March 2019 and June 2019. Participants were recruited to take part via social media and asked to choose their preferred option of 2 hypothetical health apps to prescribe to a hypothetical patient or to prescribe neither. Choices were characterized by differing levels of patient age, cost, published evidence bases, whether they had a National Health Service (NHS) stamp of approval, personal familiarity with the technology, and whether they were recommended by a fellow HCP. The results were analyzed using a mixed logit model, with subgroup analyses to account for heterogeneity.

**Results:**

We received 230 responses, a total of 96.5% (n=222/230) of respondents understood the survey task and passed the test of rationality. The median age was between 36 and 45 years, and 62.6% (n=139/222) of the health care providers responding to the survey had previously recommended the use of health apps to patients. Health apps were most likely to be prescribed to patients if they had an NHS stamp of approval or if they were recommended by another HCP (both *P*<.001). Published studies detailing clinical effectiveness were important (*P*<.001), but it would take five published studies to have the same impact on prescribing behavior as an NHS stamp of approval and two studies to be as convincing as having used the technology personally. Increasing patient age and costs resulted in significant reductions in digital health prescribing (*P*<.001), none more so than among allied health professionals. Willingness-to-pay for health apps increased by £124.61 (US $151.14) if an NHS stamp of approval was present and by £29.20 (US $35.42) for each published study. Overall, 8.1% (n=18/222) of respondents were reluctant to use health apps, always choosing the I would prescribe neither option, particularly among older HCPs, nurses, and those who do not use health apps personally. Subgroup analyses revealed significant differences in preferences among HCPs of differing ages and clinical backgrounds.

**Conclusions:**

An NHS stamp of approval, published studies, and recommendations from fellow HCPs are significant facilitators of digital prescribing, whereas increasing costs and patient age are significant barriers to engagement. These findings suggest that demonstrating assurances of health apps and supporting both the dissemination and peer-to-peer recommendation of evidence-based technologies are critical if the NHS is to achieve its long-term digital transformation ambitions.

## Introduction

Increasing public expectations of National Health Service (NHS) care, a rapidly aging population, and an ever-increasing prevalence of long-term and comorbid conditions mean that health care systems are working harder than ever. Adapting the way patients and health care professionals (HCPs) communicate and collaborate in promoting health and well-being is, therefore, essential if the future expectations of high-quality patient-centric care are to be realized.

New digital technologies, which are generally low cost and widely accessible to 95% of adults in the United Kingdom who own a smartphone [1], have the potential to be a valuable strategic tool in this transition. These technologies have the potential to deliver significant improvements in disease management by empowering patients to monitor and manage their long-term conditions [2], encourage the promotion of health and well-being, and provide care outside of traditional health care settings. Furthermore, the highly personalized real-world data captured by digital health technologies have the potential not only to improve service planning by aligning capacity more closely with demand [3] but also provide a first-of-a-kind opportunity to understand real-world condition management, enabling both informed decision making and the delivery of personalized care [4-6].

From 2024, patients in England will have the *right* to access digital primary care services under the NHS long-term plan [7]. By the end of the 10-year period covered by the plan, it is envisaged that digital care will become standard care, with people increasingly being cared for and supported at home using remote monitoring (via wearable devices), electronic services, and digital tools. Although the rollout of e-services including the electronic prescription service (EPS) has widely been a success, with 93% of England’s 7300 general practitioner (GP) practices enrolled and more than 67% of prescriptions delivered via EPS [7], clinical engagement with digital health technologies, despite a suggested appetite for digital health [8-10], is to date minimal [11,12]. A survey of nurses in Catalonia demonstrated that only 6.5% of those included frequently recommended digital technologies as part of routine patient care [11], with similar studies in France [13], Ireland [14], Norway [15], Germany [16], Belgium [17], and Australia [18] also highlighting a hesitance among health professionals to engage with and recommend digital health technologies in routine practice.

Being the gatekeepers of health services, understanding the perspectives of HCPs is crucial to achieving the long-term plans of the NHS for digital transformation, and the effective delivery and uptake of safe and high-quality digital solutions, which have proven to be clinically effective. However, to date, a paucity of evidence regarding HCP preferences for digital health solutions limits our ability to realize any potential such technologies may deliver.

On the basis of the combined results of a qualitative pilot study [19] and a targeted literature review [8-18,20,21], we conducted a discrete choice experiment (DCE) among 222 HCPs in the United Kingdom to determine priorities, trade-offs, and willingness-to-pay (WTP) for several common characteristics of digital health technologies.

## Methods

### Ethics Approval and Consent to Participate

The study did not require ethical approval as the form of the study was opinion seeking for the purpose of market research, with the subject matter limited to topics that are strictly within the professional competence of the participants. In addition, no vulnerable groups were included, the data were completely anonymous, there was no risk of disclosure, and the data collected were not sensitive in nature. We also submitted an inquiry to the NHS Health Research Authority, which confirmed that ethical approval was not required.

### Availability of Data and Materials

The data that support the findings of this study are available from the authors upon reasonable request.

### Face-to-Face Discussions and Ranking Exercise

We followed methodological guidelines from the International Society for Pharmacoeconomics and Outcomes Research [22]. First, a pragmatic literature review was conducted to identify stated preferences regarding the use of digital health. There were no time or geographical restrictions applied. The search terms used are provided in Multimedia Appendix 1. Subsequently, we invited 10 HCPs from the Northwest of England to participate in face-to-face discussions, of which 8 agreed. We asked respondents a single question, *“*What matters to you when considering the use of health-apps with your patients?” Combining the findings with the results of our targeted literature review, we created a ranking exercise provided in Multimedia Appendix 2. In total, 30 HCPs were invited via email and social media to take part in the ranking exercise, of which 28 accepted the invitation (93% participation rate). During the ranking exercise, 9 attributes, each identified through the initial face-to-face discussions and literature review, were ranked on a scale from 1 (most important) to 9 (least important) by a cohort of 85 HCPs from the United Kingdom. In addition to the face-to-face discussions, we also invited HCPs to provide responses on the web, of which 57 HCPs from the United Kingdom contributed. Further details of this exercise are provided elsewhere [19]. Following the conclusion of the ranking exercise, the 6 highest ranked attributes were selected for inclusion in the discrete choice experiment (DCE).

### Discrete Choice Experiment

A DCE, designed to elicit the preferences of HCPs for digital health prescribing, and including the attributes identified during the literature review, face-to-face discussions, and ranking exercise [19], was provided to HCPs between March and June 2019 (sample survey instrument is provided in Multimedia Appendix 3). The DCE survey was provided digitally, using Google Forms (Google, Alphabet Inc) and disseminated using social media (LinkedIn, Twitter, and Facebook). All participants consented to participation by submitting the survey on the web after being provided with a study information sheet. Demographic information was collected for all respondents. DCE methodology is well described [23-25], and given the increasing emphasis on shared decision making and value-based care, it is used extensively to measure the preferences of both patients and HCPs for the delivery of health care. In DCEs, respondents are given a hypothetical scenario, typically comparing one option to another, and asked to choose which they prefer. This process is repeated with the values (levels) of the characteristics (attributes) randomly changing each time. The choices respondents make can be used to infer preferences for each level of each of the attributes included. In our DCE, all attributes and levels were identified during the face-to-face discussions and ranking exercises and are provided in Table 1.

All respondents were HCPs working in the United Kingdom. We included qualified nursing and medical staff of all grades (nurse, GP, secondary care physician, and allied health professional), from primary, secondary, and tertiary care settings. We stated clearly in the description of the survey and advertisements that the survey was only to be answered by HCPs based in the United Kingdom; however, we also included a screening question as the first question of the survey, where respondents were asked to provide their job title. Responses from those who were not HCPs were removed at this point. Each respondent received 16 discrete choice tasks plus a test of rationality to gauge their understanding of the survey. In the test of rationality, one option was clearly superior to the alternative in every domain, including a lower price, a greater number of studies, and an NHS stamp of approval. If respondents failed the test of rationality, by selecting the inferior option, their responses were excluded from the formal analysis. Each question asked respondents to choose between 2 digital health technologies, each characterized by different levels of the attributes included (Multimedia Appendix 3), in addition to an opt-out *I would prescribe neither* option. This option was important to highlight those who were consistently reluctant to engage with digital health technologies, regardless of the characteristics of the technologies under consideration. As the full factorial design, whereby each respondent is provided with each possible combination of levels for each attribute, would have necessitated (3^3^×4^3^=1728) choices per respondent, a D-optimal design was used with 2 blocks of 16 possible questions. This approach was used to maximize statistical power while minimizing the cognitive burden to respondents. The surveys were written in language that could be understood by a 10-year old. The DCE was pilot tested in a sample of 10 HCPs not involved in the main study to gauge interpretation and response times, during which period a researcher was available to answer any questions. Minor changes were subsequently made to clarify wording and graphics.

**Table 1:** Attributes and levels of the discrete choice experiment.

Attribute	Levels
Number of studies concerning safety and effectiveness	0, 1, 2, or 3
Does the app have an NHS^a^ stamp of approval?	No or yes
Cost of the technology to the NHS, £	0, 5, 25, or 75
You have personally used the app yourself?	No or yes
Age of the patient (years)	18, 35, 55, or 75
The app has been recommended by other HCPs^b^	No or yes

^a^NHS: National Health Service.

^b^HCPs: health care professionals.

### Data Analysis

We used a mixed logit model to estimate preferences for the different levels of attributes associated with digital health technologies, thereby determining which increased or decreased utility and subsequently increased/decreased the likelihood of recommending these technologies to patients. Dummy coding was used for all categorical variables, with the number of published studies, patient age, and the cost of the app coded as linear continuous variables. We first estimated the main effects model and then estimated the effects for subgroups based on factors such as years of experience as an HCP, current digital engagement, and clinical role (eg, GP, secondary care physician, and allied health professional). WTP analyses were performed to determine how HCPs were willing to trade-off one attribute for another. CIs for WTP estimates were estimated via joint distributed bootstrapping. All analyses were performed using Stata 14 (StataCorp LP) and deemed statistically significant at the 5% level (ie, *P*<.05). Although sample size calculations represent a technical challenge in DCEs, we used Johnson and Orme’s approach [26] to estimate our minimum sample size, which was equal to 42 respondents per block.

## Results

### Characteristics of Participants

Between March 2019 and June 2019, 250 responses were received, of which 20 were excluded because of not being from HCPs. A further 8 respondents were excluded because of failing the test of rationality, suggesting an understanding rate of 96.5% (n=222/230), and a complete dataset of 222 respondents. Table 2 demonstrates the demographics of those completing the DCE.

**Table 2:** Characteristics of health care professionals completing the discrete choice experiment (N=222).

Characteristic		Value, n (%)
**Age (years)**		
	<26	5 (2.3)
	26-35	34 (15.3)
	36-45	74 (33.3)
	46-55	76 (34.2)
	56-65	27 (12.2)
	66-75	5 (2.3)
	>75	1 (0.5)
**Have you used health apps personally?**		
	Yes	169 (76.1)
	No	53 (23.9)
**Have you used health apps with patients?**		
	Yes	139 (62.6)
	No	83 (37.4)
**Role**		
	Allied health professional	86 (38.7)
	Community caregiver	5 (2.3)
	Dentist	3 (1.4)
	General practitioner	32 (14.4)
	Nurse	27 (12.2)
	Pharmacist	1 (0.5)
	Secondary care physician	40 (18)
	Other	28 (12.6)

### Barriers and Facilitators to Health Care Professionals Prescribing Digital Health Technologies

In the discrete choice analysis, all attributes were statistically different from 0, suggesting importance with respect to digital health prescribing and the decision to prescribe to patients. Table 3 illustrates the net effect on the preferences of HCPs for each characteristic. A positive coefficient represents a facilitator, and a negative coefficient represents a barrier to digital health prescribing.

Having a stamp of approval from the NHS was the most important factor in encouraging mobile health (mHealth) prescribing (β=2.36, 95% CI 2.08-2.64), followed by a recommendation from a fellow HCP (β=1.28, 95% CI 1.07-1.49) and having used health apps personally (β=1.04, 95% CI 0.83 to 1.26). Although having published studies to demonstrate safety and effectiveness was important (β=.55, 95% CI 0.44-0.67), it would take 5 published studies to be as convincing as an NHS stamp of approval and 3 to be as convincing as a recommendation from a fellow HCP. Patient age (β=−0.02, 95% CI −0.01 to −0.02) and the cost of the app (β=−0.02, 95% CI −0.02 to −0.02) were both statistically significantly associated with a reduced likelihood of prescribing digital health technologies, suggesting that as patient age (per year) and cost (per £1) increase, prescribing of digital health technologies can be expected to fall. Finally, the opt-out option was also statistically significant; regardless of a high number of clinical studies, recommendations by HCPs, or having a stamp of approval, 8.1% (n=18/222) of respondents chose to prescribe neither app, suggesting a reluctance to utilize health apps among a considerable number of respondents.

**Table 3:** Preferences for digital health technologies as reported by health care professionals.

Attribute	Coefficient (β), mean (SD)	95% CI
NHS^a^ stamp of approval (yes)	2.36^b^ (1.51)	2.09-2.64
Health app recommended by HCP^c^ (yes)	1.28^b^ (0.76)	1.07-1.49
Used health apps personally (yes)	1.04^b^ (0.82)	0.83-1.26
Published study (per additional study)	.555^b^ (0.29)	0.44-0.67
Patient age (per additional year)	−0.018^b^ (−0.01)	−0.02 to −0.01
Cost (per additional £1)	−0.019^b^ (−0.02)	−0.02 to −0.02
Alternative specific constant	1.25^b^ (1.58)	0.84-1.65
Observations	10,656 (N/A^d^)	N/A
Log likelihood	−1226.3 (N/A)	N/A

^a^NHS: National Health Service.

^b^Significant at 5% level. The table represents beta coefficients and CIs from mixed logit regression. The regression coefficients for each attribute level represent the mean part-worth utility of that attribute level in the respondent sample. A positive value denotes utility/satisfaction, and a negative value denotes disutility/dissatisfaction.

^c^HCP: health care professional.

^d^Not applicable.

### Differences in the Preferences of Health Care Professionals for Digital Health Prescribing: Subgrouping by Clinical Role and Digital Familiarity

Multimedia Appendix 4 demonstrates the varying perceptions toward digital health prescribing among HCPs with varying clinical roles, whereas Multimedia Appendix 5 breaks down perceptions by digital familiarity and current use of digital health prescribing.

Increasing patient age had a stronger negative impact on digital prescribing among allied health professionals when compared with secondary care physicians and GPs, whereas among nurses, patient age did not impact digital health prescribing behavior at all. Similarly, a recommendation to use digital health prescribing from another HCP was shown to be highly influential in promoting digital prescribing among nurses and secondary care physicians, but significantly less so among GPs and allied health professionals (Table 4), which provides the relative attribute importance for each attribute and for each subgroup of HCPs under consideration and shows that an NHS stamp of approval, a single published study, and a recommendation from other HCPs were 25, 21, and 17 times more likely to impact digital prescribing than patient age among nurses.

Having an NHS stamp of approval was the most influential factor in promoting digital prescribing across all subgroups. Having published studies to demonstrate safety and clinical effectiveness was also important to respondents, and exceptionally so among younger professionals and those unfamiliar with prescribing digital health technologies to patients. However, such studies were less important to allied health professionals who believe that cost is the most important factor in the decision to prescribe or not to prescribe health apps to patients.

Finally, reluctance to use health apps varied considerably among the HCP groups under analysis. Older clinicians (aged >46 years), nurses, allied health professionals, and those who do not currently use health apps personally, all things being equal, are far more likely to opt out of prescribing digital health technologies to patients. Conversely, the most digitally enabled groups were secondary care physicians, GPs, and those who use digital health technology to manage their own health and well-being (Multimedia Appendix 5).

**Table 4:** Relative attribute importance^a^.

Group		Value, n	Characteristics of health apps					
			Published study	NHS^b^ stamp of approval	Cost	Used health apps personally	Patient age	Recommended by another HCP^c^
All		222	7	10	6	4.4	4.3	5.4
**Use health apps personally**								
	Yes	169	7.2	10	5.8	4.8	4.6	5.5
	No	53	7.5	10	3.9	1.6	3.1	3.9
**Previously prescribed health apps to patient**								
	Yes	139	6.2	10	6.3	4.5	4.3	4.8
	No	83	8.8	10	6	3.5	3.3	5.1
**Age** **(years)**								
	>46	113	5.8	10	7.1	4	4.1	5.2
	<46	109	9.1	10	5.4	4.6	5.3	5.4
**Role**								
	General practitioner	32	6.8	10	5.6	2.2	3.6	3
	Allied health professional	86	5.1	10	6.2	4.3	6.3	4.7
	Secondary care physician	40	6.7	10	2.6	6.3	5.8	7.4
	Nurse	27	8.3	10	0.5	3.3	0.4	6.6

^a^Standardized relative attribute importance (RAI) for each attribute was calculated across the subgroups to allow for across subgroups comparisons. First, an RAI was calculated for each attribute by taking the difference between the most and least preferred levels. The RAI was then standardized across subgroups by dividing it by the RAI of the most important attribute across the subgroups (NHS stamp of approval) and multiplying it by 10. The resulting number indicates the relative importance of each attribute across the subgroups (where a higher number indicates a relatively more important attribute).

^b^NHS: National Health Service.

^c^HCP: health care professional.

### Trade-Offs: Willingness-To-Pay for the Attributes of Digital Health Technologies

HCPs expressed a WTP of £124.61 (US $152.02) for a digital health technology with an NHS stamp of approval; however, this varied from £119.14 (US $145.35) among those already familiar with prescribing digital health technology to patients, up to a maximum of £1616 (US $1971.45) among nurses, as shown in Table 5. The fact that nurses are willing to trade-off up to £1616 (US $1971.45) of NHS funds for a digital health technology with an NHS stamp of approval signifies the relatively low importance placed on cost and the high ranking of a stamp of approval by nurses. Respondents were willing to pay £29.20 (US $35.62) for each published study of effectiveness or safety, which again varied from £20.79 (US $25.36; allied health professionals) to £449.50 (US $548.37; nurses). Finally, HCPs had a WTP of £67.30 US $82.10) for digital health technologies that came recommended by other HCPs, varying from £39.84 (US $ 48.60) among GPs, to £1061.50 (US $1294.99) among nurses. Using the WTP estimates provided, it is possible to estimate WTP for various types of digital health technologies with varying features. For example, a technology with no stamp of approval that is recommended by a fellow HCP and has a single study behind it would have a WTP of (£124.61 [US $152.02]×0)+(£67.30 [US $82.10])+(£29.20 [US $35.62]×1)=£96.50 (US $117.73). Similarly, a health app with an NHS stamp of approval, with an evidence base consisting of 3 studies, but which is not recommended by a HCP would have a WTP of (£124.61 [US $152.02]×1)+(£67.30 [US $82.10]×0)+(£29.20 [US $35.62]×3)=£212.21 (US $258.89).

**Table 5:** Willingness-to-pay by health care professional subgroup.

Group		Value, n	Willingness-to-pay according to characteristics of health apps, £ (US $)			
			Published study	NHS^a^ stamp of approval	Used health apps personally	Recommended by another HCP^b^
All		222	29.20 (35.62)	124.61 (152.02)	54.81 (66.87)	67.30 (82.10)
**Use health apps personally**						
	Yes	169	31.11 (37.95)	129.44 (157.91)	62.11 (75.77)	71.28 (86.96)
	No	53	48.56 (59.24)	194.38 (237.14)	31.19 (38.05)	76.44 (93.25)
**Previously prescribed health apps to patients**						
	Yes	139	24.48 (29.86)	119.14 (145.35)	53.43 (65.18)	57.57 (70.23)
	No	83	36.89 (45.00)	125.42 (153.01)	43.37 (52.91)	64.11 (78.21)
**Age (years)**						
	>46	113	20.25 (24.70)	105.00 (128.10)	41.75 (50.93)	54.21 (66.13)
	<46	109	42.35 (51.67)	140.00 (170.79)	64.59 (78.80)	76.29 (93.07)
**Role**						
	General practitioner	32	30.28 (36.94)	134.44 (164.01)	29.56 (36.06)	39.84 (48.60)
	Allied health professional	86	20.79 (25.36)	121.38 (148.08)	52.79 (64.40)	57.38 (70.00)
	Secondary care physician	40	63.50 (77.47)	284.75 (347.38)	178.13 (217.31)	209.75 (255.89)
	Nurse	27	449.50 (548.37)	1616.00 (1971.45)	526.00 (641.70)	1061.50 (1294.99)

^a^NHS: National Health Service.

^b^HCP: health care professional.

## Discussion

### Principal Findings

In this first-of-its-kind study, examining the barriers and drivers of digital health prescribing among a broad sample of HCPs from the United Kingdom, we found that the factors most influential in digital prescribing behaviors are an NHS stamp of approval, a published evidence base, cost of the technology, and recommendation by other HCPs. Respondents expressed a WTP of more than £100 for technologies with a stamp of approval from the NHS and were willing to pay approximately an extra £30 for every additional published study. The strength of this preference varied significantly among our heterogenous cohort, influenced by clinical role, age, and current level of digital literacy. This suggests that a one-size-fits-all approach to increasing digital health adaptation is unlikely to be successful in increasing the uptake of digital health in routine practice.

A recommendation from a HCP can go a long way in encouraging patients to use digital health technology. Although previous research suggests there is an appetite for digital health technologies among HCPs [8-10], engagement is minimal [11,12], with 2 studies in Catalonia and Belgium, respectively, giving utilization rates of 0% [11] and 33% [17], and others from across the globe suggesting a similar picture [13-16]. Our finding that 62.6% of respondents have to date recommended digital health technology to patients in routine practice and that 92% would rather prescribe a digital health technology than not is an interesting and atypical finding in the context of existing literature. One explanation may be the recent advocation for digital medicine by the English pharmaceutical regulator, the National Institute for Health and Care Excellence (NICE), such as recommending digital technology as a first-line treatment for children with mild depression [27] and increasing advocacy for digital prescribing from patient groups and charities, such as Age UK’s recommendation for digital services for adults experiencing from loneliness [28].

The latter is of interest, given the strong tendency of our sample to reduce digital prescribing as patient age increased, in line with a recent study conducted among chronic obstructive pulmonary disorder specialists in Canada [21]. This behavior prevails despite evidence suggesting that older individuals are increasingly accessing digitally enabled services [29-32]. If these findings are indicative of the underlying preferences of HCPs, additional effort may be required to promote digital engagement in elderly patients, particularly among allied health professionals, secondary care physicians, and younger HCPs (<46 years), where a negative impact of increasing patient age was most significant.

We also found that the age of a HCP may also predict digital engagement, with those over the age of 46 years considerably more likely to opt out of providing digital health technology, all things being equal, than any other group. A mixed methods cross-sectional study conducted in the Czech Republic observed a similar pattern [20]. The findings of these studies combined suggest that older professionals, much like older members of the general population [33], may be more skeptical of mHealth and may, therefore, require additional support to ensure the NHS long-term digital vision becomes a reality. Combined with nurses and those who do not currently use health apps personally, if the NHS digital strategy is to become a reality, these groups could represent an ideal target for workforce development. Examples include continuous professional development, the development of specialist digital skills, such as those provided by NHS Digital’s digital academy, and the creation of digital champions, which have previously been shown to be highly effective in expanding and supporting the use of digital health technologies in routine practice [34,35].

A 2019 study of Irish chronic obstructive pulmonary disorder specialists found a need for a strong evidence before considering the adaptation digital health technology in clinical practice, with published studies seen as a surrogate for safety [14], whereas findings similar to that from this study were observed among Dutch dermatologists [10], Norwegian GPs [15], and Belgian family physicians [17]. The same was observed in this study. However, despite the assertion from regulators that mHealth randomized controlled trials (RCTs) are not the only method available for evaluating digital health technologies [36,37], part of the preference for published studies that we observed may have been rooted in a desire for *gold-standard* evidence in the form of an RCT. A limitation of our study is that we did not specify which *type* of evidence was provided with health apps, which some respondents may have assumed to be RCTs, as opposed to naturalistic studies, case-control, or qualitative studies. Each of these study types is now considered acceptable forms of evidence for the *right type* of health app under NICE’s evidence standards framework. However, it is unclear whether this notion of *not all apps being equal* [19] is accepted by HCPs who have the ultimate responsibility for patient well-being. This is a question that future research should aim to address before developers of health apps to invest too heavily in evidence generation, particularly considering the WTP estimates suggested by this analysis (£29.20 extra for each additional study and £124.61 for an NHS stamp of approval). Advocates of digital health such as the Organisation for the Review of Care and Health Applications (ORCHA), NHS Digital, NICE, and the Medicines and Healthcare Products Regulatory Agency are likely to play a key role in this process, ensuring that HCPs are well informed of the benefits that health apps and digital health more broadly can deliver, know-how and where to access technologies that have been proven to be effective, and are aware of what level of evidence is appropriate for each kind of technology available.

Our pilot study suggested that HCPs value an NHS stamp of approval above all else [19], a finding echoed in a study among Catalonian nurses [11]. This was also observed in this analysis, with every group of HCPs, regardless of age, professional role, or personal digital literacy, prioritizing this characteristic above all else. This finding, although both consistent and significant, should be interpreted with caution, as it is not abundantly clear what respondents of the survey consider as an NHS stamp of approval, which could vary from inclusion on a clinical commissioning group website, NHS choices or the NHS apps library, or inclusion within Egton medical information systems or SystmOne. Historically, it has been a difficult task for developers to achieve inclusion in the NHS apps library, and to date, only 76 digital health technologies have been listed. This may be explained by a misalignment in the evidentiary requirements for these technologies, which until recently have applied a one-size-fits-all approach to evidence, making approval difficult. As existing assessment processes such as the NHS Digital Assessment Portal begin to incorporate proportionate evidentiary requirements [36-38], these *stamps of approval* may be expected to increase. However, it is uncertain whether this is what HCPs value and, ultimately, whether this will have any impact on the digital prescribing behaviors of HCPs. Further research should aim to identify precisely what an NHS stamp of approval should look like. It is also important that we understand how this can be conveyed to HCPs to provide reassurance that technologies with an NHS stamp of approval are both safe and effective.

Our study has several strengths. First, to the best of our collective knowledge, the combination of qualitative pilot testing followed up by the quantitative assessment of relative preferences using the DCE methodology make this research the first-of-its-kind when considering attitudes toward digital health. Second, the low rate of failures during the rationality test suggests that participant understanding of the survey was very high, with just 3.5% failing to progress. This lends support for the responses received being the true beliefs of the HCPs involved, rather than stochastic variation, which can be expected in the event of misunderstanding the survey. Finally, although our participants could be considered an anomalous group of highly digitally engaged HCPs, our subgroup analyses, which adjusted for digital familiarity and personal perceptions around the use of digital technologies, found no significant differences between those who personally use health apps and those who do not, and more importantly, those who currently recommend health apps to patients and those who do not. Therefore, the findings of this study can be considered representative of underlying preferences as vignettes from all levels of digital engagement were included.

The findings of our study should also be viewed in the context of several limitations. First, although we rigorously followed methodological guidelines for eliciting preferences, our study was not large enough to capture all relevant attributes that factor into the decision to provide or not to provide health apps to patients. For example, a systematic review conducted in 2018 highlighted that excessive data creation and burden in the analysis would be a significant deterrent to GPs recommending health apps to patients [12]. Similarly, a survey of Belgian family physicians suggested that additional time taken to download and explain the technologies to patients may limit the ability of HCPs to recommend health apps [17]. As neither of these attributes were included in this study, our results cannot be considered definitive, and further research is required to understand the role of these attributes relative to those examined in this study. Second, the sample sizes for our subgroup comparisons were limited, which makes robust, precise conclusions surrounding the respondents’ WTP or comparisons between subgroups difficult. Third, a stamp of approval may, in practical terms, be many things, from a listing on NHS choices to recommendations via local trust websites or an actual NHS badge provided on app stores such as Google Play, App Store, and ORCHA. There is currently no standard for an NHS stamp of approval, and further research is required on this subject to determine how this may look in practice. Similarly, as mentioned previously, our lack of clarity regarding the details of what we meant by a published study, and the fact that not all studies in mHealth are RCTs, may have led people to value this more highly than if explained in greater detail. Finally, although stated preference techniques are a useful tool in understanding the perspectives of HCPs, nothing provides greater insights than real-world utilization. This study should, therefore, be seen as indicative until a sufficient body of real-world evidence has been generated that can adequately demonstrate facets of health apps used most commonly in routine practice.

### Conclusions

This is the first study to quantitatively determine factors associated with health app prescribing among HCPs in the United Kingdom. The findings suggest that having an NHS stamp of approval, published studies, and recommendations to use digital health technology from fellow HCPs are the greatest facilitators of digital prescribing among HCPs, whereas increasing costs and patient age are significant barriers to engagement. These findings suggest that demonstrating assurances and supporting both the dissemination and peer-to-peer recommendation of evidence-based technologies that meet health challenges are critical if the NHS is to achieve its digital transformation ambitions.

## Appendix

Multimedia Appendix 1 Search terms for literature review.

Multimedia Appendix 2 Ranking exercise to determine most important characteristics of health-apps to health care professionals.

Multimedia Appendix 3 Sample survey instrument.

Multimedia Appendix 4 Variation in health care professional preferences for digital health prescribing, subgrouped by age and clinical role.

Multimedia Appendix 5 Variation in health care professional preferences for digital health prescribing, subgrouped by digital familiarity and literacy.

## Abbreviations

DCE: discrete choice experiment
EPS: electronic prescription service
HCP: health care professional
mHealth: mobile health
NHS: National Health Service
NICE: National Institute for Health and Care Excellence
ORCHA: Organisation for the Review of Care and Health Applications
RCT: randomized controlled trial
WTP: willingness-to-pay

## References

[1] (2017). Deloitte US.

[2] Whitehead L, Seaton P (2016). The effectiveness of self-management mobile phone and tablet apps in long-term condition management: a systematic review. J Med Internet Res.

[3] Imison C, Castle-Clarke S, Watson R, Edwards N (2016). The Nuffield Trust.

[4] Chung AE, Sandler RS, Long MD, Ahrens S, Burris JL, Martin CF, Anton K, Robb A, Caruso TP, Jaeger EL, Chen W, Clark M, Myers K, Dobes A, Kappelman MD (2016). Harnessing person-generated health data to accelerate patient-centered outcomes research: the crohn's and colitis foundation of America PCORnet patient powered research network (CCFA partners). J Am Med Inform Assoc.

[5] McCabe C, McCann M, Brady AM (2017). Computer and mobile technology interventions for self-management in chronic obstructive pulmonary disease. Cochrane Database Syst Rev.

[6] Chan YY, Wang P, Rogers L, Tignor N, Zweig M, Hershman SG, Genes N, Scott ER, Krock E, Badgeley M, Edgar R, Violante S, Wright R, Powell CA, Dudley JT, Schadt EE (2017). The asthma mobile health study, a large-scale clinical observational study using ResearchKit. Nat Biotechnol.

[7] (2019). The NHS Long Term Plan.

[8] Karduck J, Chapman-Novakofski K (2018). Results of the clinician apps survey, how clinicians working with patients with diabetes and obesity use mobile health apps. J Nutr Educ Behav.

[9] Berkowitz CM, Zullig LL, Koontz BF, Smith SK (2017). Prescribing an app? Oncology providers' views on mobile health apps for cancer care. JCO Clin Cancer Inform.

[10] Ariens LF, Schussler-Raymakers FM, Frima C, Flinterman A, Hamminga E, Arents BW, Bruijnzeel-Koomen CA, de Bruin-Weller MS, van Os-Medendorp H (2017). Barriers and facilitators to ehealth use in daily practice: perspectives of patients and professionals in dermatology. J Med Internet Res.

[11] Mayer MA, Blanco OR, Torrejon A (2019). Use of health apps by nurses for professional purposes: web-based survey study. JMIR Mhealth Uhealth.

[12] Gagnon M, Ngangue P, Payne-Gagnon J, Desmartis M (2016). m-Health adoption by healthcare professionals: a systematic review. J Am Med Inform Assoc.

[13] El Amrani L, Engberink AO, Ninot G, Hayot M, Carbonnel F (2017). Connected health devices for health care in French general medicine practice: cross-sectional study. JMIR Mhealth Uhealth.

[14] Slevin P, Kessie T, Cullen J, Butler MW, Donnelly SC, Caulfield B (2020). Exploring the barriers and facilitators for the use of digital health technologies for the management of COPD: a qualitative study of clinician perceptions. QJM.

[15] Fagerlund AJ, Holm IM, Zanaboni P (2019). General practitioners' perceptions towards the use of digital health services for citizens in primary care: a qualitative interview study. BMJ Open.

[16] Safi S, Thiessen T, Schmailzl KJ (2018). Acceptance and resistance of new digital technologies in medicine: qualitative study. JMIR Res Protoc.

[17] Mutebi I, Devroey D (2018). Perceptions on mobile health in the primary healthcare setting in Belgium. Mhealth.

[18] Byambasuren O, Beller E, Glasziou P (2019). Current knowledge and adoption of mobile health apps among Australian general practitioners: survey study. JMIR Mhealth Uhealth.

[19] Leigh S, Ashall-Payne L (2019). The role of health-care providers in mhealth adoption. Lancet Digit Health.

[20] Klocek A, Šmahelová M, Knapová L, Elavsky S (2019). GPs' perspectives on ehealth use in the Czech Republic: a cross-sectional mixed-design survey study. BJGP Open.

[21] Alwashmi MF, Fitzpatrick B, Davis E, Gamble J, Farrell J, Hawboldt J (2019). Perceptions of health care providers regarding a mobile health intervention to manage chronic obstructive pulmonary disease: qualitative study. JMIR Mhealth Uhealth.

[22] Hauber AB, González JM, Groothuis-Oudshoorn CG, Prior T, Marshall DA, Cunningham C, IJzerman MJ, Bridges JF (2016). Statistical methods for the analysis of discrete choice experiments: a report of the ISPOR conjoint analysis good research practices task force. Value Health.

[23] Tinelli M, Ryan M, Bond C (2016). What, who and when? Incorporating a discrete choice experiment into an economic evaluation. Health Econ Rev.

[24] Soekhai V, de Bekker-Grob EW, Ellis AR, Vass CM (2019). Discrete choice experiments in health economics: past, present and future. Pharmacoeconomics.

[25] Louviere J, Hensher D, Swait J (2000). Stated Choice Methods: Analysis and Applications.

[26] de Bekker-Grob EW, Donkers B, Jonker MF, Stolk EA (2015). Sample size requirements for discrete-choice experiments in healthcare: a practical guide. Patient.

[27] (2019). The National Institute for Health and Care Excellence.

[28] (2017). Age UK Mobility.

[29] Burch S, Preston C, Bateup S, Hina F (2017). The use of internet-enabled cognitive behavioral therapy in the treatment of depression and anxiety amongst older people. J Aging Soc Change.

[30] Bol N, Helberger N, Weert JC (2018). Differences in mobile health app use: a source of new digital inequalities?. Inf Soc.

[31] Espie CA, Kyle SD, Williams C, Ong JC, Douglas NJ, Hames P, Brown JS (2012). A randomized, placebo-controlled trial of online cognitive behavioral therapy for chronic insomnia disorder delivered via an automated media-rich web application. Sleep.

[32] Espie CA, Emsley R, Kyle SD, Gordon C, Drake CL, Siriwardena AN, Cape J, Ong JC, Sheaves B, Foster R, Freeman D, Costa-Font J, Marsden A, Luik AI (2019). Effect of digital cognitive behavioral therapy for insomnia on health, psychological well-being, and sleep-related quality of life: a randomized clinical trial. JAMA Psychiatry.

[33] Gordon NP, Crouch E (2019). Digital information technology use and patient preferences for internet-based health education modalities: cross-sectional survey study of middle-aged and older adults with chronic health conditions. JMIR Aging.

[34] Lennon MR, Bouamrane M, Devlin AM, O'Connor S, O'Donnell C, Chetty U, Agbakoba R, Bikker A, Grieve E, Finch T, Watson N, Wyke S, Mair FS (2017). Readiness for delivering digital health at scale: lessons from a longitudinal qualitative evaluation of a national digital health innovation program in the United Kingdom. J Med Internet Res.

[35] Terry J, Davies A, Williams C, Tait S, Condon L (2019). Improving the digital literacy competence of nursing and midwifery students: a qualitative study of the experiences of NICE student champions. Nurse Educ Pract.

[36] (2019). National Institute for Health And Care Excellence.

[37] Nicholas J, Boydell K, Christensen H (2016). mHealth in psychiatry: time for methodological change. Evid Based Ment Health.

[38] Greaves F, Joshi I, Campbell M, Roberts S, Patel N, Powell J (2019). What is an appropriate level of evidence for a digital health intervention?. Lancet.

