# Supplementary material for: Barriers and Facilitators to the Adoption of Mobile Health Among Health Care Professionals From the United Kingdom: Discrete Choice Experiment
Source: JMIR Mhealth Uhealth. 2020 Jul 6;8(7):e17704. doi: 10.2196/17704 (PMC7381009; doi:10.2196/17704)
Supplement: Multimedia Appendix 1 [file mhealth_v8i7e17704_app1.pdf]

APPENDIX 1: SEARCH TERMS FOR LITERATURE REVIEW

MEDLINE Search: Searched 16th September 2019

**1**	mHealth	**18**	perception*
**2**	eHealth	**19**	belief*
**3**	m-health	**20**	attitude*
**4**	mobile health	**21**	***OR/ 13-20***
**5**	smartphone app*	**22**	doctor*
**6**	digital health	**23**	nurse*
**7**	cellphone app*	**24**	healthcare provider*
**8**	cell phone app*	**25**	health-care provider*
**9**	health-app*	**26**	clinician*
**10**	health app	**27**	physician*
**11**	digital technolog*	**28**	GP*
**12**	***OR/ 1-11***	**29**	general practitioner
**13**	discrete choice experiment	**30**	general practice
**14**	discrete-choice experiment	**31**	professionals
**15**	conjoint analysis	**32**	***OR/ 22-31***
**16**	stated preference	**33**	***AND/ 12, 21, 32***
**17**	preference*		
