# Supplementary material for: Barriers and Facilitators to the Adoption of Mobile Health Among Health Care Professionals From the United Kingdom: Discrete Choice Experiment
Source: JMIR Mhealth Uhealth. 2020 Jul 6;8(7):e17704. doi: 10.2196/17704 (PMC7381009; doi:10.2196/17704)
Supplement: Multimedia Appendix 2 [file mhealth_v8i7e17704_app2.pdf]

SUPPLEMENTARY FIGURE 1

Please rank the following attributes from **1 (most important)** to **9 (least important)** in influencing your decisions to prescribe a health-app to patients

Attribute	Ranking (1 most important, 9 least important)
The app has an NHS stamp of approval, CE mark or other indicator of quality assessment	
There are published studies demonstrating the safety/effectiveness of the app	
The cost of the app	
Having access to appropriate IT for app testing/demonstrating (including internet, devices)	
You have used/checked the app yourself	
The app has been created by a clinician/health professional or clinical body	
The app is easy to prescribe (It is listed in EMIS/System One)	
The app has been recommended by other clinicians/royal college/clinical network	
Unmet need in the condition the app is designed to treat (e.g. long waiting lists with mental health, lack of follow-up care)	
