# Supplementary material for: Barriers and Facilitators to the Adoption of Mobile Health Among Health Care Professionals From the United Kingdom: Discrete Choice Experiment
Source: JMIR Mhealth Uhealth. 2020 Jul 6;8(7):e17704. doi: 10.2196/17704 (PMC7381009; doi:10.2196/17704)
Supplement: Multimedia Appendix 3 [file mhealth_v8i7e17704_app3.pdf]

SUPPLEMENTARY FIGURE 2

Scenario 1/16

	Option A	Option B	I wouldn't prescribe either
Number of studies concerning safety or effectiveness	0	3	
Does the app have an NHS stamp of approval?	No	Yes	
Cost to the NHS	£75	£0	
You have used the app yourself	Yes	No	
Age of the patient	75	18	
The app has been recommended by other clinicians	Yes	No	
**I choose....**	☐	☐	☐

Scenario 2/16

	Option A	Option B	I wouldn't prescribe either
Number of studies concerning safety or effectiveness	2	1	
Does the app have an NHS stamp of approval?	Yes	No	
Cost to the NHS	£75	£0	
You have used the app yourself	No	Yes	
Age of the patient	18	75	
The app has been recommended by other clinicians	Yes	No	
**I choose....**	☐	☐	☐

Scenario 3/16

	Option A	Option B	I wouldn't prescribe either
Number of studies concerning safety or effectiveness	1	2	
Does the app have an NHS stamp of approval?	No	Yes	
Cost to the NHS	£5	£25	
You have used the app yourself	No	Yes	
Age of the patient	35	55	
The app has been recommended by other clinicians	Yes	No	
**I choose....**	☐	☐	☐

ORCHA™
Organisation for the Review of Care & Health Applications
