# Supplementary figures and images for: Barriers and Facilitators to the Adoption of Mobile Health Among Health Care Professionals From the United Kingdom: Discrete Choice Experiment
Source: JMIR Mhealth Uhealth. 2020 Jul 6;8(7):e17704. doi: 10.2196/17704 (PMC7381009; doi:10.2196/17704)

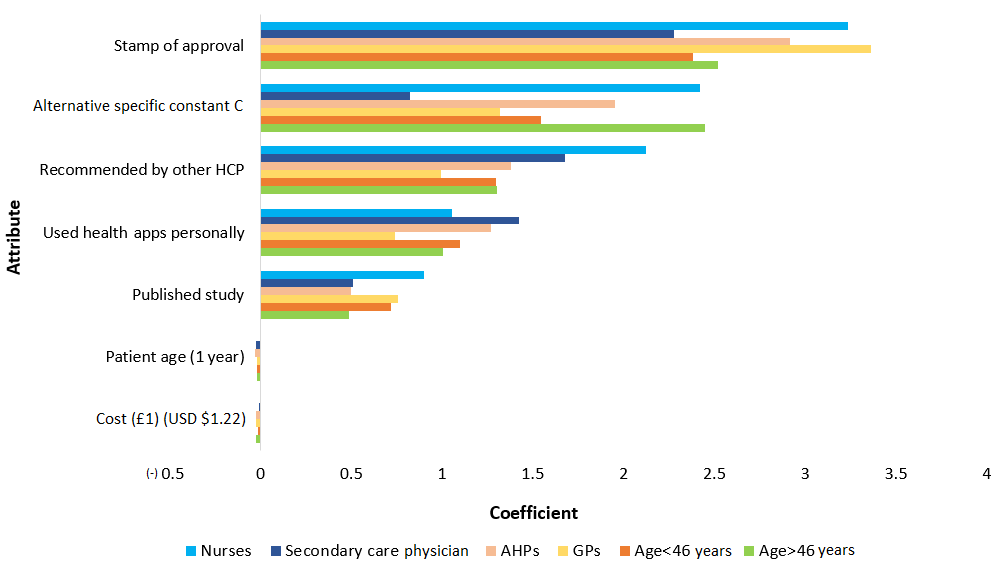

Supplement: Multimedia Appendix 4 [file mhealth_v8i7e17704_app4.png]

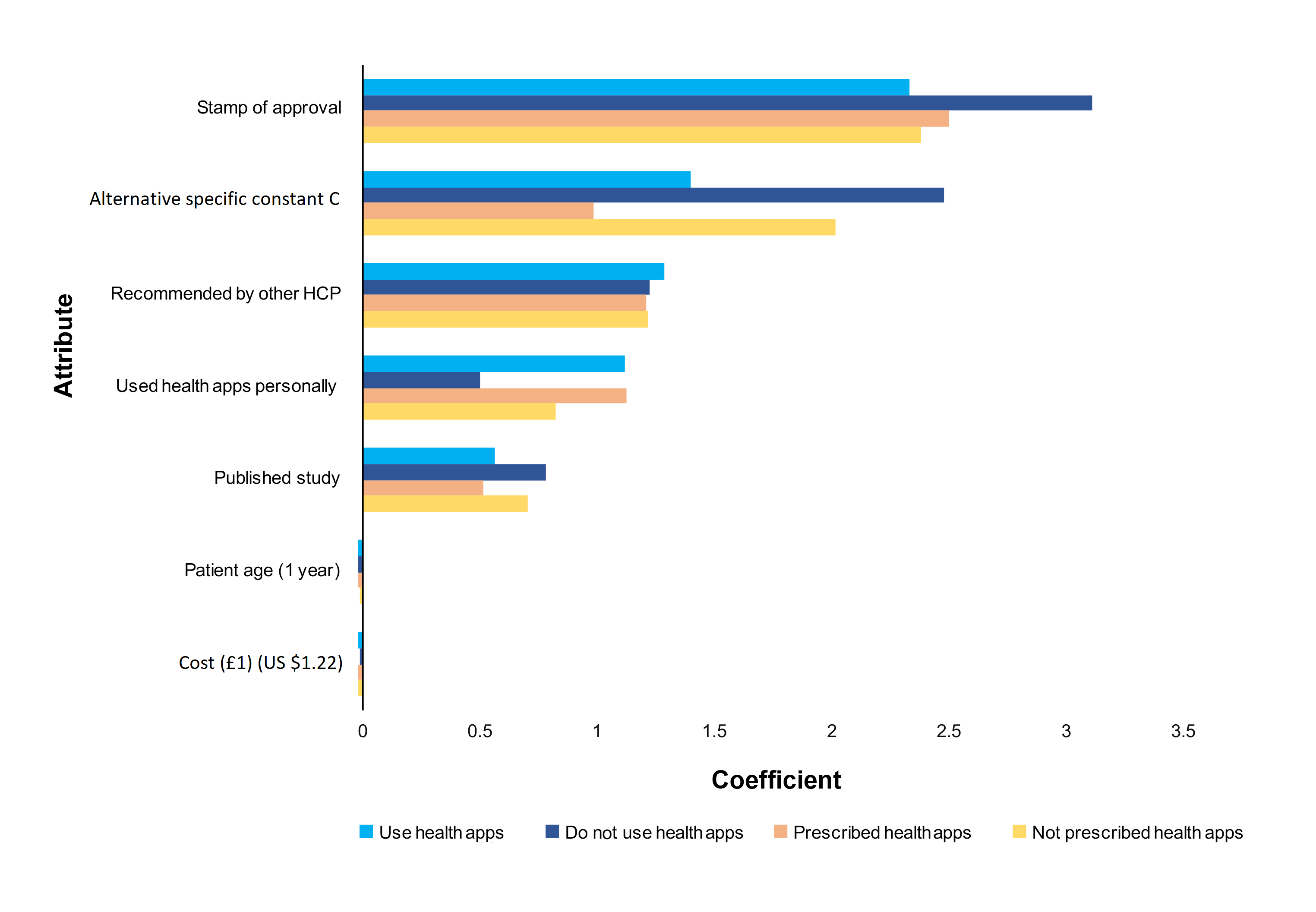

Supplement: Multimedia Appendix 5 [file mhealth_v8i7e17704_app5.png]
